# Are physical activity and/or adherence to the Mediterranean diet determinants of the changes found in kinanthropometric variables, body composition and physical fitness in adolescents?

**DOI:** 10.1186/s12887-024-04796-x

**Published:** 2024-05-20

**Authors:** Adrián Mateo-Orcajada, Raquel Vaquero-Cristóbal, María del Mar Sánchez-Serrano, Lucía Abenza-Cano

**Affiliations:** 1 Facultad de Deporte, UCAM Universidad Católica de Murcia, Murcia, Spain; 2 Department of Physical Activity and Sport, Faculty of Sport Sciences, University of Murcia, Murcia, Spain; 3 IES Maristas La Sagrada Familia, Cartagena, Murcia, 30203 Spain

**Keywords:** Body composition, Nutrition pattern, Physical fitness, Physical activity level, Youth

## Abstract

The practice of physical activity and adherence to the Mediterranean diet (AMD) have been extensively studied for their relationship with kinanthropometric, body composition and physical fitness variables. However, no previous study has analyzed whether these healthy habits are equally determinant for the differences found in kinanthropometric, body composition and physical fitness variables or, on the contrary, if one of them is more relevant. For this reason, the objectives of the present study were: (1) to analyze the differences in kinanthropometric, body composition, and physical fitness variables between adolescents with different levels of physical activity and AMD, and (2) to determine whether physical activity and/or AMD are predictors of differences in kinanthropometric variables, body composition or physical fitness in adolescents. The sample consisted of 791 adolescents (404 males and 387 females; mean age: 14.39±1.26 year-old) whose physical activity level, AMD, kinanthropometric variables, body composition and physical fitness, were measured. The results showed differences when considering the level of physical activity in kinanthropometric variables, body composition and physical fitness, but not the level of AMD, which was relevant only when it was poor, and the adolescents were inactive. Nevertheless, the AMD did not seem to exert such a determining effect as to produce significant differences on its own. On the other hand, the practice of physical activity did act as a predictor mainly of changes in the fitness variables. Therefore, the main novelty of the present study is the establishment of an order of importance of the healthy habits acquired by adolescents, concluding that the practice of physical activity is more determinant for the differences found in the study variables.

## Introduction

The acquisition of healthy lifestyle habits during adolescence is essential for the proper development of the adolescent population [1, 2]. These habits have a high probability of being maintained during adulthood [1, 2], preventing the development of numerous chronic diseases and the loss of autonomy and quality of life [3, 4]. The practice of physical activity and nutrition are among the healthy habits that have acquired greater importance in recent decades due to their impact on health [5, 6]. For this reason, they have been widely implemented in programs aimed at improving the physical and mental health of the adolescent population [7, 8].

This is because the practice of physical activity has numerous health benefits for adolescents [9]. Among the most relevant are the improvement of kinanthropometric, body composition and fitness variables. In this regard, a decrease in variables related to fat mass, and the increase in muscle mass have been observed; as well as an improvement in cardiorespiratory fitness, sit-ups, and upper limb resistance tests [10]. Moreover, this improvement in the physical fitness of adolescents is associated to an improvement in sports performance in many sport modalities such as football, alpine ski, or tennis [11–13]. These benefits could also lead to adolescents staying longer in sport [14].

Similarly, the acquisition of adequate nutritional habits is fundamental in this population [15]. Adolescents who do not skip any meals and have a higher consumption of fruit and vegetables, as well as a lower consumption of sugary drinks, show a better health status. Thus, better psychological development has been observed [16]; also optimal kinanthropometric variables, characterized by an increase in skeletal muscle mass and a lower accumulation of fat mass [17, 18]; as well as better performance in physical fitness tests in these adolescents [19]. In particular, previous research has focused on a specific nutritional pattern, the adherence to the Mediterranean diet (AMD). This pattern has been shown to be one of the best structured nutritional recommendations and to include numerous health-related nutritional habits [20, 21]. Thus, previous research has demonstrated the relationship between AMD, kinanthropometric variables and physical performance. In this regard, adolescents with better AMD had lower fat accumulation, waist circumference and body mass index (BMI) [22]. Similarly, these adolescents had higher physical performance in cardiorespiratory tests [23], thus establishing itself as one of the key nutritional patterns for the adolescent population.

In addition to the individual benefits of physical activity and AMD for the adolescent population, a positive relationship has been observed between both healthy habits, with adolescents who engage in more physical activity having a greater AMD [24]. Previous research has shown significant differences in kinanthropometric, body composition and fitness variables according to the level of physical activity and AMD [10, 22, 23]. However, no previous research has analyzed the differences in neither the kinanthropometric variables, nor in body composition, nor in the physical fitness variables, in adolescents with the same level of AMD but different levels of physical activity, nor among adolescents with different levels of AMD but the same level of physical activity. Thus, it is not clear whether one of the habits exerts a greater influence on the differences previously found. Only one study is known to have shown that adolescents from different sports modalities, for which the level of physical activity performed was not controlled, with a greater AMD, showed a lower fat mass and better physical performance [25]. However, as inactive adolescents were excluded from the study, it is not known whether the differences were due to their AMD or to the effects from regular physical activity, and future research is needed to corroborate these results in active and inactive adolescents.

Based on the results of previous research, it is proposed as a research hypothesis that adolescents with a higher level of physical activity, regardless of their level of AMD, as well as adolescents with better AMD, regardless of their level of physical activity, will present better kinanthropometric variables and body composition, as well as better performance in physical fitness tests, than inactive adolescents and those with lower levels of AMD. If the present hypothesis is accepted, it would be demonstrated that both healthy habits are determinant and influential in the differences in kinanthropometric variables, body composition and physical fitness of adolescents.

In order to contrast this hypothesis, the objectives set for the present investigation were: (1) to analyze the differences in kinanthropometric, body composition, and physical fitness variables between adolescents with different levels of physical activity and AMD; and (2) to determine whether physical activity and/or AMD are predictors of differences in kinanthropometric variables, body composition or physical fitness in adolescents.

## Materials and methods

### Design

A cross-sectional study was conducted to determine differences in kinanthropometric, body composition and physical fitness variables in a sample of adolescents with different levels of AMD and physical activity. The study lasted for one year (from June 2021 to May 2022) during which adolescents from four different compulsory secondary schools were assessed. Prior to the start of the study, the institutional ethics committee of the Catholic University of Murcia (code: CE022102) approved the research design, in accordance with the World Medical Association codes and the Helsinki declaration. In addition, the study design as well as the development of the manuscript followed the STROBE statement [26].

### Participants

Sampling was non-probabilistic by convenience, with the minimum sample size calculated with Rstudio statistical software (v.3.15.0; Rstudio Inc., Boston, MA, USA) using standard deviations (SD) from previous research based on adolescent physical activity (SD = 0.58) [27] and AMD (SD = 2.32) [23]. This methodology for sample size calculation has been used in previous research [28]. Thus, the minimum sample size was 756 adolescents, assuming an error (d) of 0.05 for physical activity and 0.22 for AMD for a 99% confidence interval (CI). Considering that acceptable statistical power is greater than 0.80 [29], the statistical power of the study was calculated for the sample indicated for the physical activity and AMD variables. The calculated statistical power was 0.96, which is high.

For the present study, four compulsory secondary education schools (high schools) were selected from different geographic areas of the Region of Murcia that had the largest number of students enrolled [30]. Thus, a final sample of 791 adolescents (404 males and 387 females) aged between 12 and 16 years (mean age: 14.39±1.26) was obtained.

The education centers, adolescents, and parents were informed of the objectives and procedures of the research study, and participation was completely voluntary. The inclusion criteria were (a) not having any surgical intervention or incapacitating disease that would prevent participation in the tests; (b) age between 12 and 16 years old; (c) attending compulsory secondary education. The exclusion criteria were a) not completing all physical tests or questionnaires. The adolescents who agreed to participate and met the inclusion criteria, provided an informed consent form signed by them and their parents.

### Instruments and procedure

The measurement of the kinanthropometric and body composition variables, the performance of the physical fitness tests, and the completion of the questionnaires by the adolescents were carried out in the same day, using the covered pavilions of the compulsory secondary education centers, during the physical education class hour. Thus, the possible interference of polluting environmental variables in the results of the research was minimized.

A measurement protocol was established for data collection. First, the completion of the physical activity and AMD questionnaires; second, the measurement of the kinanthropometric and body composition variables; third, the sit-and-reach test; fourth, the warm-up, familiarization, and the execution of the handgrip strength, countermovement jump (CMJ), and 20-m sprint tests; and fifth, the 20-m shuttle run test.

#### Physical activity and AMD questionnaires

The “Physical Activity Questionnaire for Adolescents” (PAQ-A) [31] and the “Mediterranean Diet Quality Index for children and adolescents” (KIDMED) [20] were used, with both questionnaires being valid and reliable in their Spanish version [20, 32]. The PAQ-A is composed of 9 questions that refer to the physical activity performed in the previous week. It is completed with a Likert scale of 1 to 5 points (1: no physical activity; 5: a lot of physical activity). The final score of the questionnaire is obtained by averaging the scores of the first 8 items, allowing adolescents to be classified as active (≥2.75) (A) or inactive (< 2.75) (IN) [33]. The KIDMED questionnaire is composed of 16 items that are completed with a dichotomous scale (yes or no). The final score ranges from 0 to 12 points, as it has 12 questions with a positive connotation (+ 1) and four with a negative connotation (-1). The final score allows the classification of AMD as follows: poor adherence (PA) (0–3 points), need to improve adherence (NIA) (4–7 points), and optimal adherence (OA) (8–12 points) [20].

#### Kinanthropometric variables and body composition measurement

Anthropometrists (level 2 to 4), accredited by the International Society for the Advancement of Kinanthropometry (ISAK), measured the kinanthropometric variables, ensuring the quality of the measurements by following a well-defined protocol in which the error is lower the higher the level of the anthropometrist from level 1 to level 4 [34]. This assessment included two basic measurements (body mass and height), five girths (relaxed arm, waist, hips, thigh, and calf), and three skinfolds (triceps, thigh, and calf). The protocol followed for the measurements was as established by the ISAK [35]. In addition, the following derived variables were calculated from these measurements: BMI (kg/m^2^), waist-to-hip ratio (waist girth/hips girth), corrected arm girth [arm relaxed girth - (π*triceps skinfold)], corrected thigh girth [middle thigh girth - (π*thigh skinfold)], corrected calf girth [calf girth - (π*calf skinfold)], fat mass (%) [36], muscle mass [37], and sum of 3 skinfolds (Σ triceps, thigh and calf).

The following instruments were used to measure the kinanthropometric variables, as they were valid and reliable: a TANITA BC 418-MA Segmental scale (TANITA, Tokyo, Japan) for measuring body mass with an accuracy of 100 g; a stadiometer 213 (SECA, Hamburg, Germany) for measuring height, with an accuracy of 0.1 cm; a skinfold caliper (Harpenden, Burgess Hill, UK) for measuring skinfolds with an accuracy of 0.2 mm; and an inextensible tape (Lufkin W606PM, Missouri City, TX, USA) with an accuracy of 0.1 cm for measuring girths. All instruments were previously calibrated.

Each kinanthropometric variable was measured twice, with a third measurement being necessary when the difference between the first two was greater than 5% in skinfolds and 1% in the rest of the measurements. The final value corresponded to the mean of the values, when two measurements were taken, or to the median, when three measurements were taken [35]. All measurements for each adolescent were performed by the same anthropometrist.

In a subsample, the intra- and inter-evaluator technical error of measurement (TEM) was calculated. Thus, the intra- and inter-evaluator TEM was 0.02% and 0.03% for the basic measurements, 1.21% and 1.98% for the skinfolds, and 0.04% and 0.06% for the girths, respectively.

#### Physical fitness

Four researchers were in charge of the familiarization and overseeing of the physical fitness tests, with the same investigator always being in charge of the same test during all the measurements, to avoid inter-evaluator error.

#### Sit-and-reach test

Prior to warm-up, a single attempt of the sit-and-reach test was performed to assess hamstring flexibility [38], as previous research has shown the effect that warming up, as well as the repeated execution of this test, can have on the final result of the test [39]. For this, the participants started seated, with their knees fully extended, toes facing upwards, ankles together and soles of the feet resting on an Acuflex Tester III box (Novel Products, Rockton, IL, USA), and then performed a maximum trunk flexion in which they tried to reach the maximum possible distance by sliding their hands, one over the other, along the top of the box, keeping their knees and arms fully stretched during the execution [40].

#### Warm-up, familiarization, and execution of the handgrip, CMJ, and 20-m sprint tests

The order of the rest of the physical tests was chosen complying with the recommendations from the National Strength and Conditioning Association (NSCA) on the fatigue generated and the metabolic demand of each test [41].

Based on previous research, a generic warm-up was performed that included 5 min of progressive running and 10 min of joint mobility of the main joints involved in the physical fitness tests [42]. The warm-up ended with a more specific part corresponding to the familiarization of the adolescents with the handgrip strength, CMJ, and 20-m sprint tests.

The handgrip strength, CMJ, and 20-m sprint tests were performed randomly by the adolescents, repeating each test twice with a period of 2 min between each attempt, and 5 min between each test. The best value of each test was taken as the final result. The handgrip strength test assessed the maximum handgrip strength [43], using a Takei Tkk5401 digital handheld dynamometer (Takei Scientific Instruments, Tokyo, Japan) on which the adolescents, with their elbow fully extended, had to apply the maximum possible force [44].

The CMJ test assessed the adolescents’ lower limb power by means of vertical jump using a force platform with a sampling frequency of 200 Hz (MuscleLab, Stathelle, Norway). The adolescents stood on the force platform and had to perform a 90° knee and hip flexion followed by an extension as quickly as possible to reach the maximum jump height. It should be noted that during the entire execution, the hands were to be kept on the hips and the trunk fully extended [45].

The 20-m sprint was used to measure the adolescents’ speed [46]. For this, single-beamed photocells (Polifemo light, Microgate, Italy) were used to measure the minimum time it took the adolescents to run a distance of 20 m. Based on previous research, it was determined that the ideal height for placing the photocells was at the hips, since the probability of adolescents cutting them with their arms was reduced to 4%, as compared to 60% when they were placed at chest height [47, 48]. The participants began the test by standing behind a line placed where the photocells were located and decided by themselves when to start the run at maximum speed [46].

#### 20-m shuttle run test

For measuring cardiorespiratory capacity, the 20-m shuttle run test was performed only once and at the end of all the physical fitness tests, as it is an incremental test that ends with the exhaustion of the participants [49, 50]. This is an incremental test in which adolescents must run 20 m guided by a beep sound signal indicating the intensity at which they should run. The test ends when the adolescent is not able to run the distance twice consecutively before the beep sounds, or when the adolescent reaches exhaustion. The speed at which the adolescent finishes the test is used to predict the maximal oxygen consumption (VO2max) using Léger’s formula [49].

### Data analysis

The normality of the data was assessed using the Kolmogorov-Smirnov test, as well as the study of skewness, kurtosis, and variance, showing that the data followed a normal distribution, which allowed us to apply parametric tests for their analysis. A MANOVA analysis was performed to determine the differences in kinanthropometric variables, body composition and physical fitness between active poor adherence (A-PA), active need to improve adherence (A-NIA), active optimal adherence (A-OA), inactive poor adherence (IN-PA), inactive need to improve adherence (IN-NIA), and inactive optimal adherence (IN-OA) adolescents. A subsequent Bonferroni post-hoc analysis allowed differences to be established between active adolescents with different levels of AMD, inactive adolescents with different levels of AMD, and active and inactive adolescents with the same levels of AMD. Partial eta squared (η2) was used to define the effect size (ES), defined as small (ES≥0.10), moderate (ES≥0.30), large (ES≥1.2) or very large (ES≥2.0), with an error of *p* < 0.05 [51]. A structural equation model (SEM) was used to determine the influence of physical activity and AMD on changes in kinanthropometric, body composition and physical fitness variables. A value of *p* < 0.05 was set to determine statistical significance. Statistical analyses were performed with the SPSS statistical package (v.25.0; SPSS Inc., Chicago, IL, USA).

## Results

Table 1 shows the differences in the kinanthropometric and body composition variables, as well as in the physical fitness variables between adolescents with different levels of physical activity and AMD. The differences were significant in all kinanthropometric and body composition variables, except for body mass (*p* = 0.548), height (*p* = 0.056), BMI (*p* = 0.580), waist girth (*p* = 0.670), and hips girth (*p* = 0.549), and in all the physical fitness variables, except for the sit-and-reach test (*p* = 0.469).

**Table 1:** Descriptive statistics of adolescents with different level of AMD and level of physical activity, and analysis of differences between groups

Variable	Descriptors (Mean ± SD)						F, *p*	η2
	IN-PA (*n* = 60)	IN-NIA (*n* = 243)	IN-OA (*n* = 127)	A-PA (*n* = 33)	A-NIA (*n* = 169)	A-OA (*n* = 159)		
Body mass (Kg)	55.55±10.29	56.62±13.04	57.14±14.89	53.65±10.87	57.94±13.01	57.93±13.30	0.803; *p* = 0.548	0.005
Height (cm)	164.43±8.51	162.58±8.65	162.31±8.91	161.01±7.42	164.33±9.50	164.62±9.13	2.169; *p* = 0.056	0.014
BMI (kg/m^2^)	20.51±3.27	21.33±3.96	21.53±4.56	20.50±3.23	21.43±3.80	21.27±3.67	0.758; *p* = 0.580	0.005
Waist girth (cm)	68.77±7.23	69.20±9.00	69.82±10.06	69.18±6.36	70.61±8.61	69.79±8.40	0.639; *p* = 0.670	0.004
Hips girth (cm)	89.51±7.74	91.03±9.21	91.56±9.91	88.50±9.06	90.33±9.33	90.88±8.67	0.801; *p* = 0.549	0.005
Waist to hip ratio	0.77±0.05	0.76±0.05	0.76±0.05	0.78±0.05	0.78±0.07	0.77±0.05	4.132; *p* = 0.001	0.027
Corrected arm girth (cm)	21.07±2.16	20.95±2.93	20.88±3.17	21.17±2.87	22.02±2.95	21.60±3.19	3.374; *p* = 0.005	0.022
Corrected thigh girth (cm)	39.42±3.77	39.51±4.55	39.42±5.05	39.18±4.83	41.35±4.93	41.05±5.46	4.775; *p* < 0.001	0.031
Corrected calf girth (cm)	29.12±2.32	28.95±2.76	28.99±4.08	28.97±2.98	30.14±3.63	29.59±2.94	3.284; *p* = 0.006	0.022
Fat mass (%)	21.00±8.67	24.01±10.09	25.44±11.45	20.55±7.79	20.64±10.10	22.14±10.06	4.456; *p* = 0.001	0.029
Muscle mass (kg)	18.34±3.89	18.04±4.79	17.96±5.34	18.51±4.65	20.43±5.07	19.89±5.43	6.552; *p* < 0.001	0.042
Sum of 3 skinfolds (cm)	46.52±21.23	54.86±25.27	58.17±27.83	48.30±20.36	46.23±24.03	49.60±23.99	4.749; *p* < 0.001	0.031
VO2max (ml/kg/min)	38.06±6.24	37.78±5.15	38.06±4.85	40.85±4.33	42.02±5.67	41.77±5.70	19.811; *p* < 0.001	0.118
Handgrip right arm (kg)	27.05±6.53	25.63±7.79	24.83±7.93	26.14±7.34	28.95±8.28	27.69±9.44	4.971; *p* < 0.001	0.032
Handgrip left arm (kg)	25.56±6.44	23.88±7.30	22.98±7.48	24.40±7.29	27.00±7.59	25.47±7.85	5.326; *p* < 0.001	0.035
Sit-and-reach (cm)	16.06±8.47	16.09±8.66	15.86±8.49	12.82±9.01	16.50±8.50	15.35±9.42	0.917; *p* = 0.469	0.006
CMJ (cm)	23.88±6.51	22.37±6.06	21.42±7.75	22.86±8.31	25.97±6.86	25.07±6.83	9.444; *p* < 0.001	0.060
20-m sprint (s)	3.96±0.67	4.02±0.50	4.08±0.65	3.93±0.39	3.76±0.45	3.83±0.47	8.178; *p* < 0.001	0.052

IN: inactive; A: active; PA: poor adherence to Mediterranean diet; NIA: need to improve adherence to Mediterranean diet; OA: optimal adherence to Mediterranean diet; BMI: body mass index; VO2max: maximum oxygen consumption; CMJ: countermovement jump

**Fig. 1:**
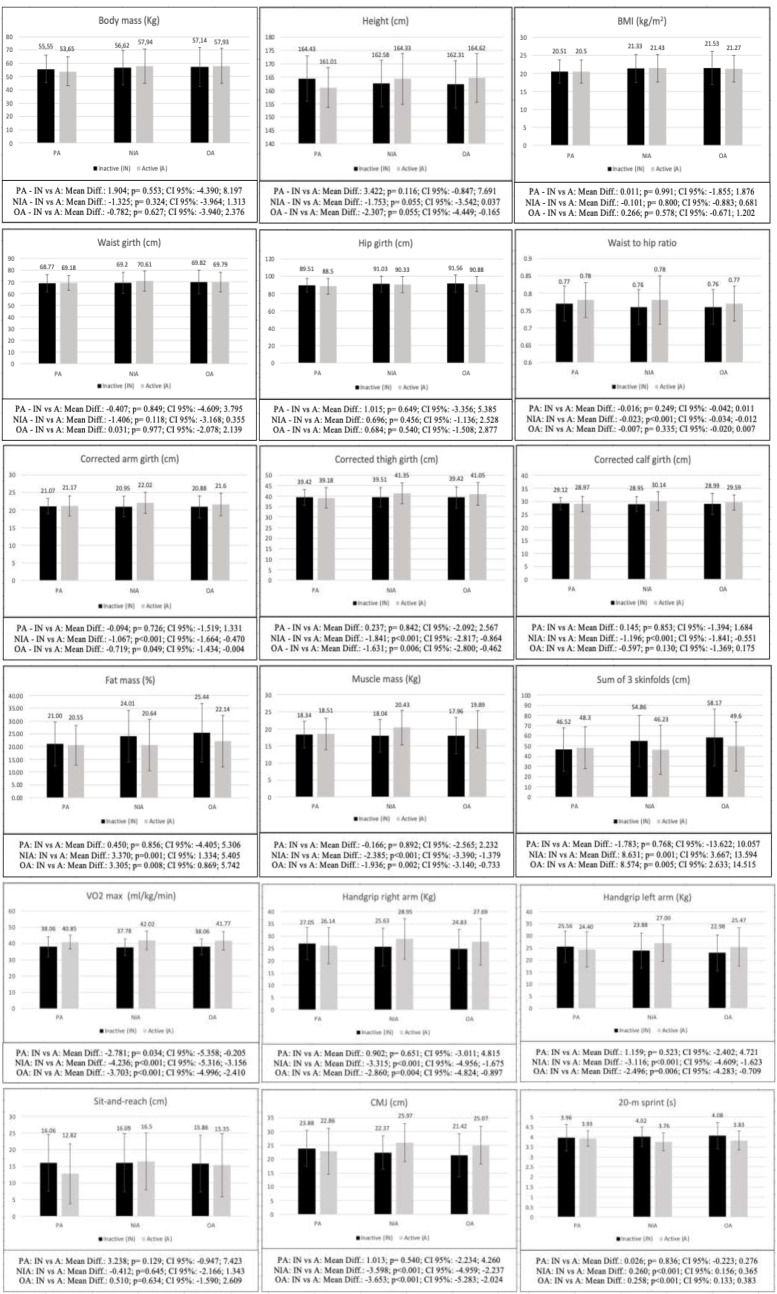
Bonferroni post-hoc analysis of the differences between adolescents with different level of physical activity and same level of AMD. BMI: body mass index; sum of 3 skinfolds: summatory of 3 skinfolds; VO2max: maximal oxygen consumption; CMJ: countermovement jump

The subsequent post-hoc analysis (Fig. 1) showed that differences between adolescents with the same levels of AMD and different levels of physical activity were present in the waist-to-hip ratio, corrected arm, thigh and calf girths, fat mass, muscle mass, sum of 3 skinfolds, VO2max, handgrip right and left arm, CMJ, and 20-m sprint. Thus, the differences were significant in all the variables mentioned in the group of adolescents with NIA (*p* < 0.001–0.001) and with OA (*p* < 0.001–0.049), except in waist-to-hip ratio (*p* = 0.335) and corrected calf girth (*p* = 0.130) in the group with OA, with the active adolescents showing higher scores in all the variables, except for fat mass and sum of 3 skinfolds. The group of adolescents with PA to the Mediterranean diet (MD) showed significant differences only in VO2max (*p* = 0.034), with active adolescents showing higher performance.

**Fig. 2:**
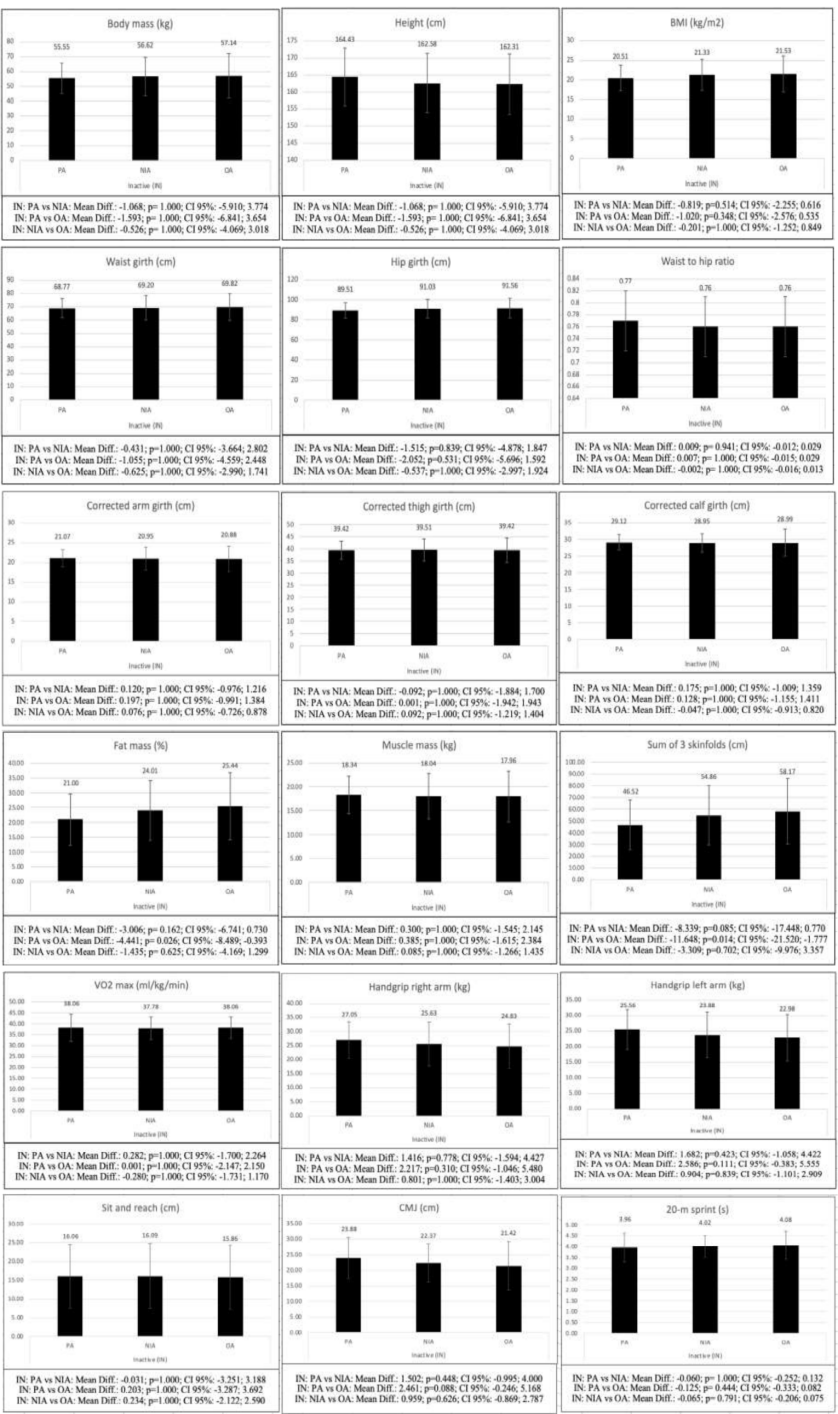
Bonferroni post-hoc analysis of the differences between inactive adolescents with different level of AMD. BMI: body mass index; sum of 3 skinfolds: summatory of 3 skinfolds; VO2max: maximal oxygen consumption; CMJ: countermovement jump

**Fig. 3:**
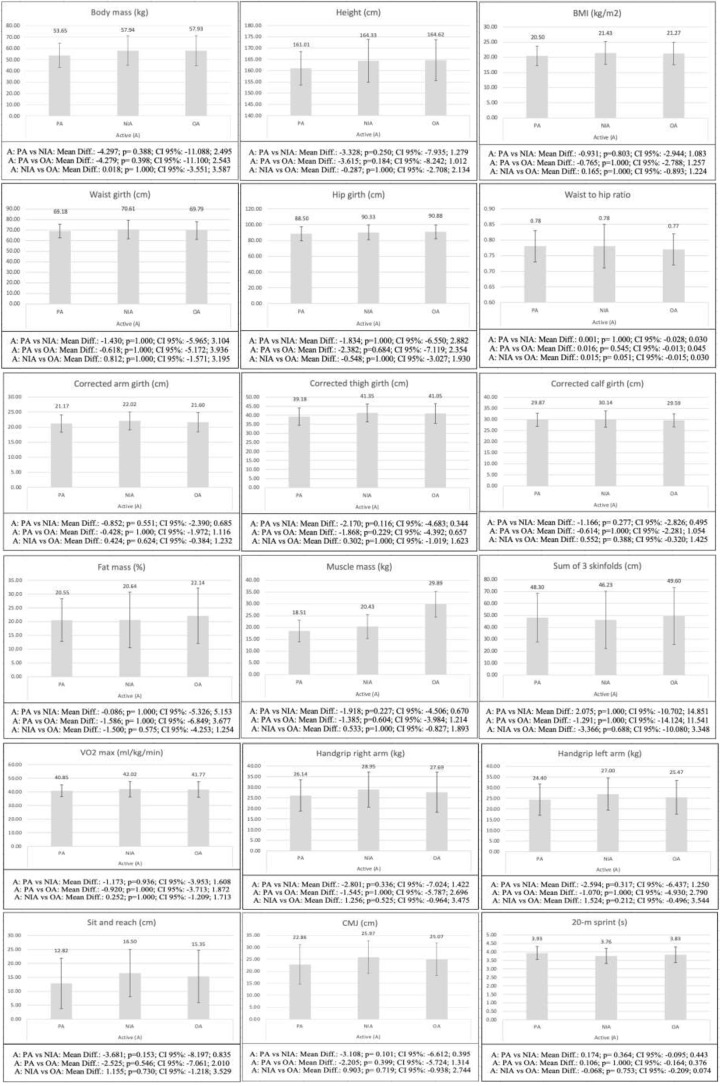
Bonferroni post-hoc analysis of the differences between active adolescents with different level of AMD. BMI: body mass index; sum of 3 skinfolds: summatory of 3 skinfolds; VO2max: maximal oxygen consumption; CMJ: countermovement jump

When comparing inactive adolescents with different levels of AMD (Fig. 2), the differences were significant in fat mass (*p* = 0.026) and sum of 3 skinfolds (*p* = 0.014) between the groups with PA and OA to the MD, with the adolescents in the OA group showing higher values in both variables. On the other hand, the comparison between active adolescents with different levels of AMD did not show statistically significant differences neither in the kinanthropometric and body composition variables (*p* = 0.051-1.000), nor in the physical fitness variables (*p* = 0.101-1.000), between any of the groups analyzed (Fig. 3).

**Fig. 4:**
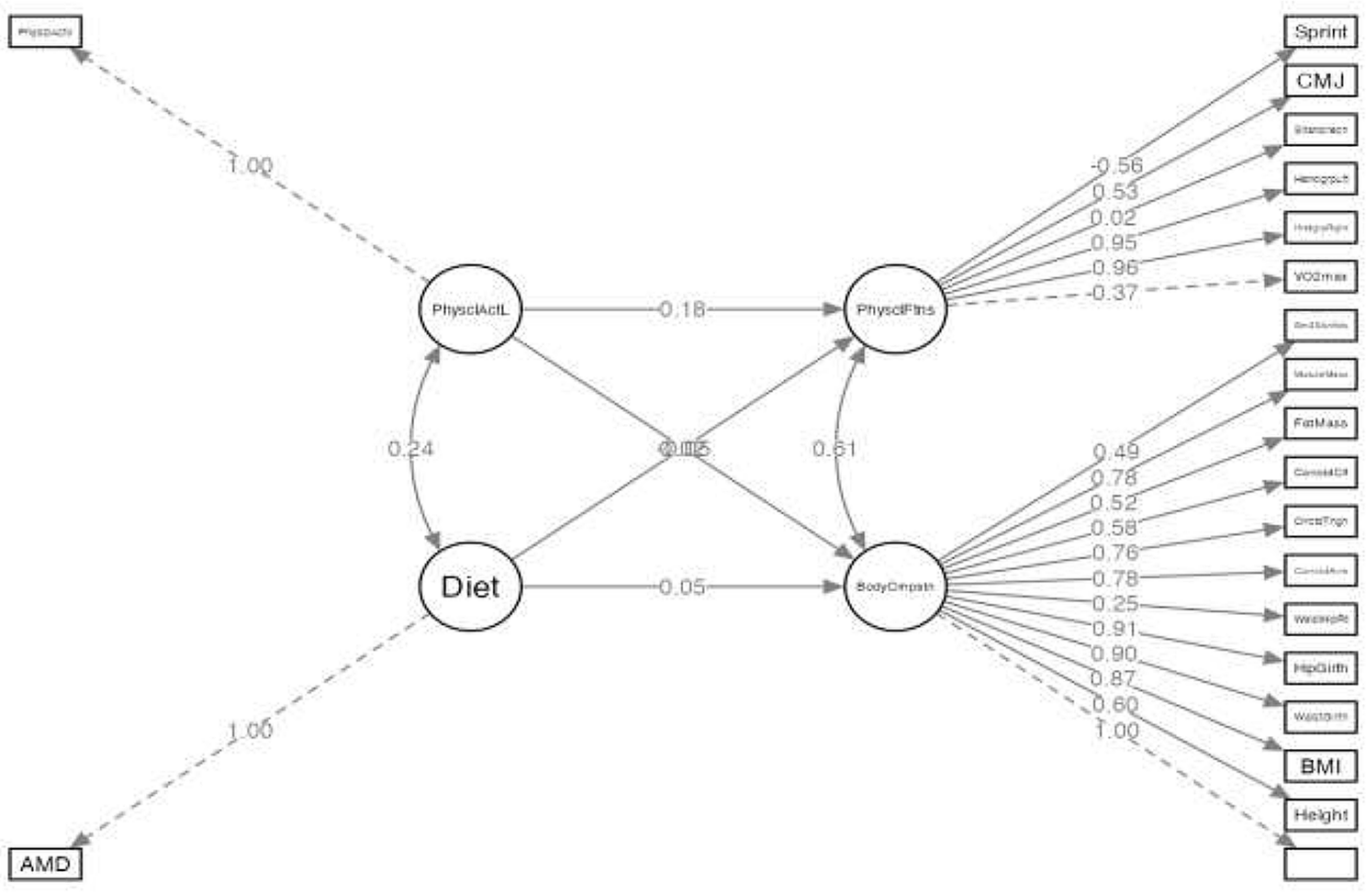
Structural equation model (SEM) for the study variables. PhysclAct: physical activity level; AMD: adherence to Mediterranean diet; PhyysclFtns: physical fitness; BodyCmpstn: body composition; CMJ: countermovement jump; handgrplft: handgrip left; handgrpRght: handgrip right; Sm3Sknfds: summatory of 3 skinfolds; CorrctdClf: corrected calf; CrrctdThgh: corrected thigh; CrrctdArm: corrected arm; WaistHipRt: waist to hip ratio

The results of the SEM showed that neither physical activity (*p* = 0.660) nor AMD (*p* = 0.204) were determinant factors for the changes that occurred in the kinanthropometric and body composition variables of the adolescents. However, in the physical fitness variables, the practice of physical activity was shown to be determinant (*p* < 0.001), but not AMD (*p* = 0.179), although the predictive capacity of the model was low (R^2^ = 0.031) (Table 2; Fig. 4).

**Table 2:** Structural equation model to analyse the impact of physical activity and AMD in the dependent variables

Dependent Variable	Independent Variable	*R* ^2^	β	Estimate	SE	95% CI	z	p
Body composition	AMD	0.002	0.048	0.262	0.206	-0.14; 0.67	1.272	0.204
	Physical activity		0.016	0.334	0.757	-1.15; 1.82	0.441	0.660
Physical Fitness	AMD	0.031	-0.051	-0.045	0.033	-0.11; 0.02	-1.343	0.179
	Physical activity		0.182	0.590	0.135	0.33; 0.85	4.379	< 0.001

AMD: adherence to the Mediterranean diet

## Discussion

The present study sought to analyze the differences in kinanthropometric, body composition, and physical fitness variables between adolescents with different levels of physical activity and AMD to establish whether both healthy habits were determinant in the changes found in these variables. In this regard, the results of the present investigation indicated that active adolescents showed higher scores in all variables compared to inactive adolescents, except for fat variables, mainly in the NIA and OA groups to the MD. When comparing only inactive adolescents with different level of AMD, the results showed that adolescents with OA showed higher fat mass accumulation than adolescents with PA. While when comparing active adolescents, no significant differences were found in any of the variables analyzed in the groups with different levels of AMD. The SEM results show how physical activity could be a determinant and predictor of changes in physical fitness variables, but no influence of any of the independent variables (physical activity and AMD) on kinanthropometric and body composition variables was found.

A first significant finding of the present research was found when analyzing the differences between active and inactive adolescents with the same AMD levels. The results showed that active adolescents with NIA and OA to the MD scored higher in corrected girths, muscle mass, VO2max, handgrip, CMJ, and sprint tests, and lower in fat mass and sum of 3 skinfolds than inactive adolescents with these same AMD levels. Therefore, in adolescents who maintain a healthy diet, the benefits of regular physical activity are enough to produce changes in kinanthropometric, body composition and fitness variables. These results are similar to those found in previous studies in which only the level of physical activity was assessed, with active adolescents showing greater muscle development and physical performance, while inactive adolescents having a greater accumulation of fat mass [10]. This may be because physical activity generates an increase in calorie expenditure, which may lead to a decrease in adiposity-related variables [52, 53]. Furthermore, physical activity could be a stimulus that generates increases in the muscle mass in adolescents [54]; and also better fitness, as a consequence of physiological, neuromuscular, and morphological adaptations in musculoskeletal system [10, 55].

However, these positive results in active versus inactive adolescents occurred only in adolescents with NIA and OA to the MD, but not in those with PA, where only differences in VO2max were found. Previous research showed similar results for VO2max where differences were found when considering the level of physical activity, regardless of gender, age, weight, or maturational status [42]. The present research provides new information in this respect, showing that active adolescents show higher VO2max, regardless of their AMD. This could be explained by the fact that the improvement in cardiorespiratory capacity is dependent on a large number of variables related to sports practice, including the number of weekly sessions, combination of aerobic and resistance training, or exercise intensity [56], but not so much on the adolescents’ diet.

Nevertheless, the absence of differences between active and inactive adolescents in kinanthropometric, body composition and fitness variables in the PA to the MD group might indicate that poor nutritional habits dissipate the benefits that regular physical activity could bring to the kinanthropometric, body composition and the majority of physical fitness variables. This could be because nutritional intake can also condition energy balance, which is directly related to kinanthropometric variables and body composition [17], but also to physical fitness, given the direct relationship between these kinanthropometric and physical fitness variables [19]. Therefore, the present research seems to indicate that if the practice of physical activity is not accompanied by an adequate diet, it does not generate enough benefits in kinanthropometric, body composition and physical fitness variables to differentiate between active and inactive adolescents, which differs from previous research in which the importance of AMD was questioned [57]. However, these previous studies have not compared the groups under the same conditions of physical activity, so the present study opens a new avenue of research in this respect.

Therefore, regarding the differences between active and inactive adolescents with different levels of AMD, it is important to highlight that physical activity becomes more relevant as long as adolescents maintain an adequate AMD. However, when adolescents have PA to MD, AMD can dissipate the changes in kinanthropometric, body composition and fitness variables that are achieved through the practice of physical activity.

A second significant finding of the present research was found when analyzing the differences between AMD levels with the same physical activity level. It was found that among inactive adolescents there were no significant differences in most of the anthropometric, body composition and physical fitness variables analyzed as a function of AMD. No previous research is known to have analyzed differences in kinanthropometric, body composition and physical fitness variables of inactive adolescents with different AMD. However, previous research has shown that adolescents with better AMD, regardless of their level of physical activity, do not have better kinanthropometric variables, body composition and physical fitness than those with worse AMD [57]. This may be because AMD alone does not have sufficient weight to generate adaptations in such parameters, as previous research has shown [57, 58]. These results coincide with those found in the present investigation where no differences were found in most of the variables analyzed as a function of AMD when analyzing inactive adolescents.

However, a surprising result of the present investigation was that in the inactive adolescents there were significant differences between the PA and OA to the MD groups in fat mass and sum of 3 skinfolds, with higher values found in the adolescents from the OA group. One possible explanation for these results could be that in the area of nutrition, a negative energy balance, mainly based on increasing total daily energy expenditure through physical activity, is essential for achieving changes in kinanthropometric parameters such as fat mass [59]. Therefore, the adherence to a nutritional pattern such as the AMD is not enough to produce changes in kinanthropometric and body composition variables if it is not accompanied by the practice of physical activity, as shown in programs that combine diet with sport practice in adolescents [60]. Another possible explanation could be related to the fact that previous research has shown that females tend to adhere better to the MD [24]. This could influence the values of fat mass and sum of 3 skinfolds, as adolescence is the stage in which females begin to accumulate adipose tissue to a greater extent than males [61–63]. Another explanation is that the AMD assessment assesses adequacy in nutritional habits to a healthy pattern, but not caloric intake [64]. Therefore, it could be that those adolescents with OA to the MD have higher caloric intakes, due to the belief that eating healthy foods is no longer important in quantity, which would influence the accumulation of adiposity [65]. However, future research is needed to corroborate these results, by performing the analysis according to gender, as well as using complementary methods to obtain information on the nutritional intake of adolescents.

Regarding the differences in active adolescents with different levels of AMD, the results showed no significant differences in any of the study variables when comparing active adolescents with different levels of AMD. These results are in line with previous research in active adolescents in which AMD was not a determinant of the changes observed in kinanthropometric, body composition and fitness variables [58, 66]. This could be due to the fact that in active adolescents diet is not as relevant to generate adaptations in anthropometric variables, body composition or physical condition because they compensate for possible deficits with regular physical activity [58, 66]. Therefore, AMD could play a relevant role, but always accompanied by other physical activity or fitness variables. This is relevant for interventions to be carried out with adolescent population, in which the joint inclusion of physical activity and AMD could be the best option, together with the consideration of other factors such as family and school environment, as has been shown in previous research [67, 68].

Structural equation modelling supports this, as it showed that physical activity alone influenced the differences found in the fitness variables. However, neither AMD alone, nor physical activity was predictive of changes in kinanthropometric and body composition variables. There is little previous research that has examined the predictive ability of physical activity and AMD to lead to changes in anthropometric variables, body composition and physical fitness. However, similar previous research to predict the development of obesity during adolescence found that the most important predictor of freedom from obesity was participation in extracurricular physical activity and limited screen use [6, 69]. Despite the results of the present study, it should also be noted that the ability to influence changes in the dependent variables was low. Therefore, the information obtained in the present investigation should be corroborated in future research and the predictive ability of physical activity and AMD on kinanthropometric, body composition and fitness variables in adolescents needs further research.

The results obtained in the present research allow partially accepting the research hypothesis. This is because the differences were significant between active and inactive adolescents with same level of AMD, with the active adolescents having better kinanthropometric and body composition variables, as well as better performance in physical fitness tests, than inactive ones, but not in the group with PA to the MD. However, when analyzing same active/inactive group with different AMD levels, it was found that inactive adolescents with OA presented greater fat accumulation. In the active adolescent group, no significant differences were found between the AMD groups. These results are important as they indicate that physical activity might influence kinanthropometric variables, body composition and fitness. However, AMD might not be as important, mainly for active adolescents, as long as they do not have poor AMD, where diet would become more relevant. Despite the results, the SEM showed that the predictive ability of physical activity is low for the study variables, and there was no AMD influence, so caution should be exercised in extrapolating the results.

With respect to the practical implications derived from the present study, it must be underlined that the practice of physical activity seemed to be the most relevant healthy habit responsible for the differences found in kinanthropometric, body composition and physical fitness result. However, the AMD was especially relevant, because when poor, it seemed to hinder the occurrence of changes in the variables analyzed. However, only a small predictive capacity of physical activity on some of the physical fitness variables analyzed was observed, so future studies should investigate which other lifestyle habits may play a relevant role to improve adolescent health.

According to the limitations of the present study, it should be noted that the state of biological maturation of the adolescents was not considered, which is extremely important in the changes found in the kinanthropometric variables and body composition, as well as in the physical fitness of the adolescents [61]. Furthermore, physical activity and AMD were measured using questionnaires which, although valid and reliable, provide limited and totally subjective information. In addition, the AMD question assesses the acquisition of healthy nutritional habits, but not caloric intake. Future research should use accelerometers for the assessment of physical activity and multi-day dietary records for nutritional intake. VO2max was measured indirectly from a field test and mathematical formulas, which could condition the assessment of the cardiorespiratory capacity of the adolescents. In addition, future research should implement long-term intervention programs that will provide more information on the role of physical activity and AMD. Finally, although the high schools with the largest number of students were selected, the sample selection was made by convenience, which should be considered when extrapolating the results.

Despite the above-mentioned limitations, the study has the following strengths (a) there is a large sample of adolescents; (b) both physical activity and AMD were measured together, as well as their influence on kinanthropometric, body composition and fitness variables; (c) more scientific evidence has been provided on the importance of each of these healthy habits on the kinanthropometric, body composition and physical fitness variables in the adolescent population; (d) body composition has been analyzed by kinanthropometric variables, including not only estimation formulas, but also skinfolds and girths that provide more data and the possibility of comparison; and (e) physical fitness has been assessed according to the most relevant tests for this population according to the scientific literature and their measurement has been carried out rigorously, repeating the tests performed to obtain greater reliability and validity of the measurements.

## Conclusion

In conclusion, the main novelty of the present study is that it allows establishing the order of importance of the practice of physical activity and AMD in the differences found in the kinanthropometric, body composition and the physical fitness results of the adolescents. In this regard, the practice of physical activity seemed to be the most determinant healthy habit, but a poor AMD played a limiting role in the changes observed between active and inactive adolescents. An optimal AMD alone did not seem to exert a sufficient effect for producing changes among adolescents with the same levels of physical activity. Furthermore, it is also important to highlight that physical activity can predict changes in fitness variables, but the predictive capacity was low.

## Data Availability

The data that support the findings of this study are available from the corresponding author, R. V-C., upon reasonable request.
